# Robust Supramolecular Dimers Derived from Benzylic-Substituted 1,2,4-Selenodiazolium Salts Featuring Selenium⋯π Chalcogen Bonding

**DOI:** 10.3390/ijms232314973

**Published:** 2022-11-29

**Authors:** Alexander A. Sapronov, Alexey A. Artemjev, Gleb M. Burkin, Victor N. Khrustalev, Alexey S. Kubasov, Valentine G. Nenajdenko, Rosa M. Gomila, Antonio Frontera, Andreii S. Kritchenkov, Alexander G. Tskhovrebov

**Affiliations:** 1 Research Institute of Chemistry, Peoples’ Friendship University of Russia, 6 Miklukho-Maklaya Street, 117198 Moscow, Russia; 2 N.D. Zelinsky Institute of Organic Chemistry, Russian Academy of Sciences, 47 Leninsky Prosp., 119334 Moscow, Russia; 3 Kurnakov Institute of General and Inorganic Chemistry, Russian Academy of Sciences, 31 Leninsky Prosp., 119071 Moscow, Russia; 4 Department of Chemistry, M.V. Lomonosov Moscow State University, 1, Leninskie Gory, 119991 Moscow, Russia; 5 Departament de Química, Universitat de les Illes Balears, Crta de Valldemossa km 7.5, 07122 Palma de Mallorca (Baleares), Spain; 6 Institute of Technical Acoustics NAS of Belarus, Ludnikova Prosp. 13, 210009 Vitebsk, Belarus

**Keywords:** selenium⋯π interactions, noncovalent interactions, chalcogen heterocycles, chalcogen bonding

## Abstract

The series of benzylic-substituted 1,2,4-selenodiazolium salts were prepared via cyclization reaction between 2-pyridylselenyl chlorides and nitriles and fully characterized. Substitution of the Cl anion by weakly binding anions promoted the formation supramolecular dimers featuring four center Se_2_N_2_ chalcogen bonding and two antiparallel selenium⋯π interactions. Chalcogen bonding interactions were studied using density functional theory calculations, molecular electrostatic potential (MEP) surfaces, the quantum theory of atoms-in-molecules (QTAIM), and the noncovalent interaction (NCI) plot. The investigations revealed fundamental role of the selenium⋯π contacts that are stronger than the Se⋯N interactions in supramolecular dimers. Importantly, described herein, the benzylic substitution approach can be utilized for reliable supramolecular dimerization of selenodiazolium cations in the solid state, which can be employed in supramolecular engineering.

## 1. Introduction

A bottom-up approach to the synthesis of functional materials with programmable structures and therefore properties is a long-standing challenge. In the past decades, coordination chemistry has been successfully employed as a tool to create such materials, which resulted in the emergence of metal-organic frameworks (MOFs) [1,2]. In analogous fashion, supramolecular organic frameworks (SOFs) [3,4] have recently emerged, which utilize noncovalent interactions as a tool for a programmable self-assembly. Among SOFs, hydrogen-bonded organic frameworks (HOFs) [4,5,6,7,8] represent a promising class of materials. However, halogen (XB) and chalcogen bonding (ChB) have recently appeared as powerful alternatives to the hydrogen bonding due to their directionality and tunability [9,10,11,12,13,14,15,16,17,18,19,20,21,22,23,24,25,26,27,28,29,30]. In spite of their potential advantages, XB and ChB have received a very limited attention for the creation of extended materials analogous to HOFs [31].

In this context, chalcogenodiazoles represent attractive objects for the creation of such materials [32,33,34,35,36,37,38]. They were shown to be capable of forming symmetrical antiparallel supramolecular dimers via two Se⋯N ChB interactions. These important supramolecular synthons were intensively studied in the last years [33,34,35,36,37,38].

Recently, we described the synthesis of cationic 1,2,4-selenodiazolium salts via unprecedented cyclization between bifunctional 2-pyridylselenyl reagents and nitriles [39,40,41,42,43,44,45]. The latter new heterocycles also showed a potency to form Se_2_N_2_ supramolecular dimers in some instances. However, for some of them, Se_2_N_2_ dimers formation was not observed when other weak noncovalent forces outcompeted the squares formation [41,42,43]. These stimulated us to search for the strategies to reliably form Se_2_N_2_ synthons.

Here, we describe the synthesis of benzylic-substituted 1,2,4-selenodiazolium salts. When halide anions were substituted by the weakly binding anions, we always observed the formation Se_2_N_2_ squares, supported by the pair of antiparallel selenium⋯arene ChB interactions. These interactions have been further studied and characterized using density functional theory (DFT) calculations, molecular electrostatic potential (MEP) surfaces, the quantum theory of atoms-in-molecules (QTAIM), and the noncovalent interaction (NCI) plot.

## 2. Results and Discussion

**Scheme 1:**
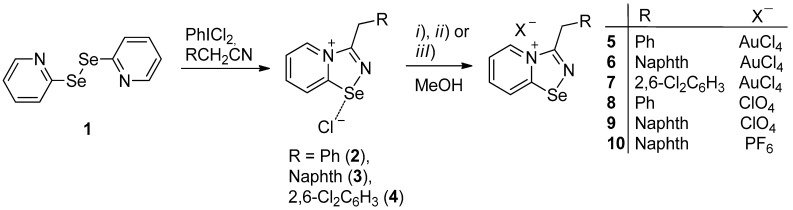
Synthesis of **2**–**10**. (*i*) NaAuCl_4_, (*ii*) LiClO_4_, (*iii*) NBu_4_PF_6_.

Benzylic-substituted 1,2,4-selenodiazolium chlorides **2**–**4** were prepared by the oxidation of diselenide **1** with PhICl_2_ and sequential cyclization of in situ generated 2-pyridylselenyl chloride with corresponding aryl acetonitriles in 78–93% yields (Scheme 1). The NMR and mass spectrometry for data for **2**–**4** were in accord with the proposed structures.

**Figure 1:**
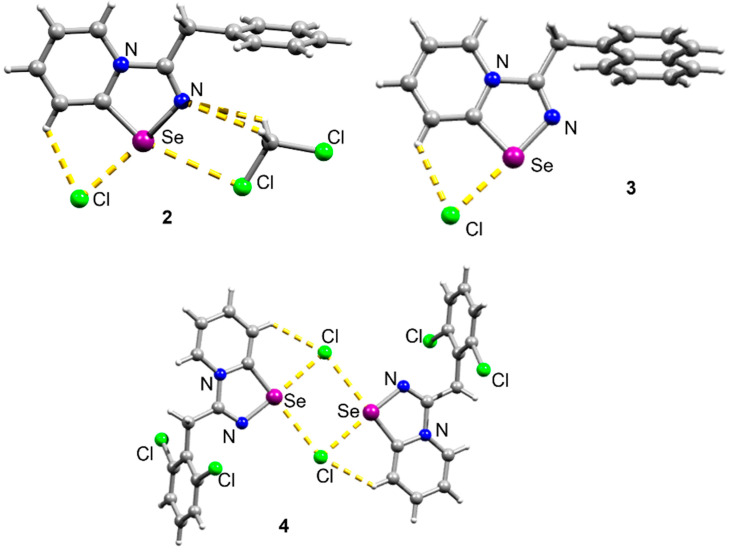
Ball-and-stick representations of crystal structures of **2**–**4**. Grey and light-grey spheres represent carbon and hydrogen, respectively.

Compounds **2**–**4** could be recrystallized from MeOH to give single crystals suitable for X-ray structural analysis, which confirmed their structures (Figure 1).

Selenodiazolium cations expectedly interacted with the Cl anions via a pair of “chelating” Se⋯Cl and H⋯Cl non-covalent interactions. Such chelation was observed earlier for all structurally characterized selenodiazolium chlorides so far [40,42,43]. Compounds **2** and **3** did not feature Se_2_N_2_ dimers in the solid state. Instead, one of the Se σ-holes in **2** was involved in Se⋯Cl ChB interaction with co-crystallized CH_2_Cl_2_ molecule, while in **3** this position was found vacant (Figure 1). In contrast, compound **4** formed Se_2_Cl_2_ dimers featuring bridging Cl anions involved in two ChB interactions.

Further, we switched to salts with weakly coordinating anions, i.e., ClO_4_^−^, AuCl_4_^−^, and PF_6_^−^. The compounds **5**–**9** were simply prepared by the addition of saturated methanolic solutions of NaAuCl_4_, NBu_4_PF_6_, or LiClO_4_ to **2**–**4** in MeOH, which resulted in the precipitation of desired compounds in 48–67% yields (Scheme 1). Analytical data (see Experimental Part) suggested the formation of selenodiazolium salts **5**–**10**.

**Figure 2:**
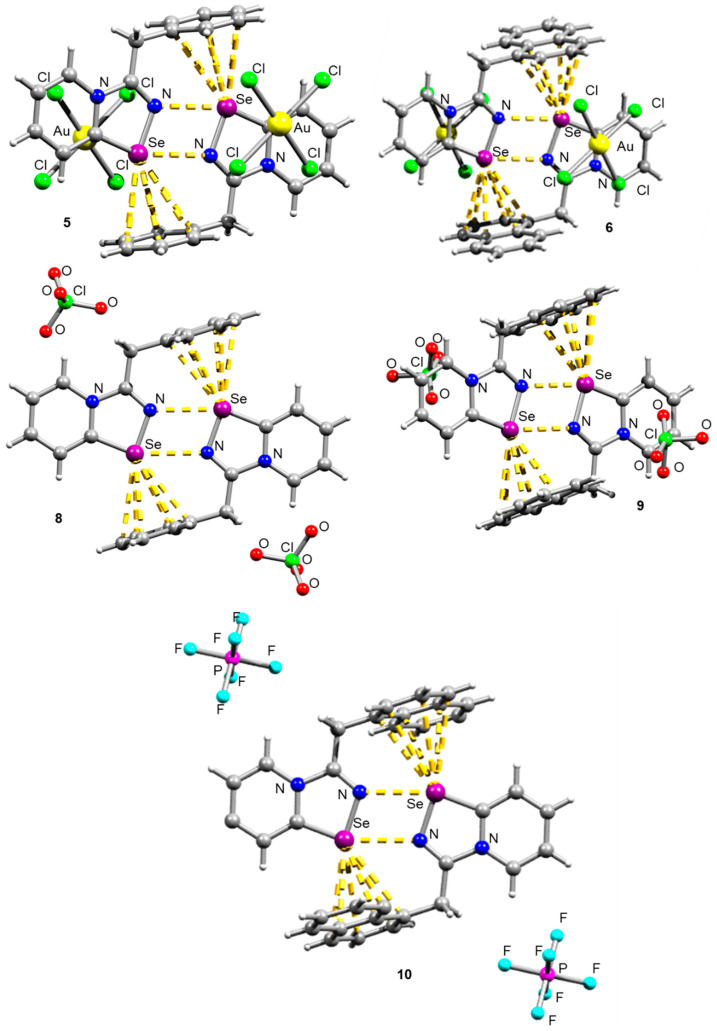
Ball-and-stick representations of crystal structures of **5**, **6**, **8**, **9**, and **10**, demonstrating supramolecular dimerization via two Se⋯N and Se⋯π ChB interactions. Grey and light-grey spheres represent carbon and hydrogen, respectively.

The structures were unambiguously established using single-crystal X-ray structural analysis (Figure 2). Bond lengths and angles are similar to what was observed earlier for selenodiazoles and other relevant heterocycles [46,47,48,49,50,51,52].

Importantly, **5**, **6**, **8**, **9**, and **10** formed supramolecular dimers in the solid state via two antiparallel Se⋯N and Se⋯π ChB interactions (Figure 2). The dimerization occurred for both benzyl and naphthylmethyl derivatives and did not depend on the nature of weakly coordinating anion.

**Figure 3:**
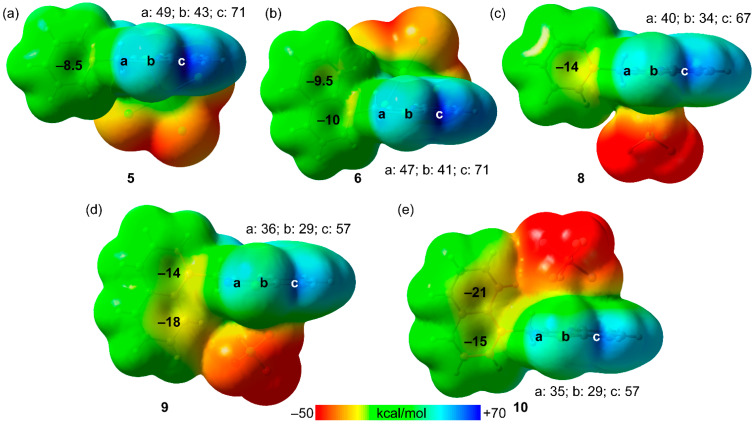
MEP surfaces of compounds **5** (**a**), **6** (**b**), **8** (**c**), **9** (**d**), and **10** (**e**) at the PBE0-D3/def2-TZVP level of theory. The values at selected points of the surfaces are given in kcal/mol.

The Se–π interactions resembled *η*^6^ metal–arene coordination complexes in terms of their geometry. To obtain more insights into Se⋯π ChB in **5**, **6**, **8**, **9**, and **10**, we performed theoretical studies starting from the calculations of the molecular electrostatic potential (MEP) surfaces using the salts to have neutral systems. The MEP surfaces are gathered in Figure 3. The values at both σ-holes (labeled **a**,**c**) and at the middle of both σ-holes (labeled as **b**) are indicated. In all cases, the σ-hole labeled as “**c**” is deeper (ranging 57 to 71 kcal/mol) than the other one (“**a**” ranging 35 to 49 kcal/mol) due to the overlap with the positive and adjacent H-atom of the pyridyl ring. The MEP values at the middle of both σ-holes are also positive (“**b**” ranging 29 to 43 kcal/mol), thus suggesting that the directionality of the Se⋯π interaction is not crucial. Regarding the aromatic rings, the MEP surfaces reveal that the values are negative, ranging from −8.5 in **5** to −21 kcal/mol in **10**. The larger negative MEP values in the aromatic rings of **9** and **10** are due to the closer proximity of the counterion.

**Figure 4:**
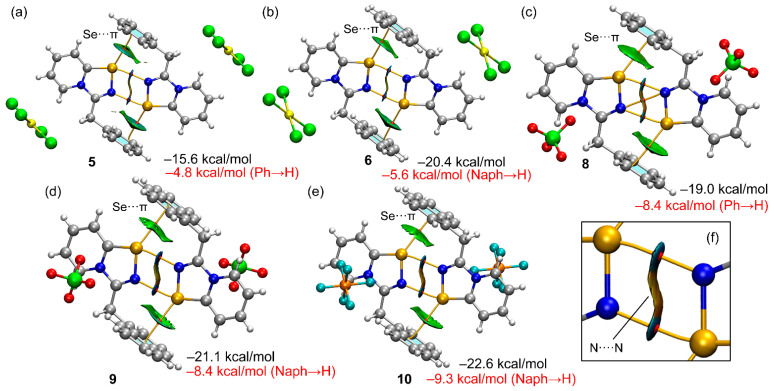
Combined QTAIM (CPs in red and bond paths as orange lines) and NCIPlot analyses of the dimers of **5** (**a**) **6** (**b**), **8** (**c**), **9** (**d**), and **10** (**e**) and an amplification of the Se⋯N interaction (**f**). The dimerization energies using the X-ray geometries and the mutated dimers are indicated using black and red font, respectively. Isovalue for the RDG isosurface 0.45.

The combined QTAIM/NCIplot analyses of the self-assembled dimers of the compounds shown in Figure 3 were computed and are represented in Figure 4 along with the dimerization energies. These dimerization energies were computed considering the salt as the monomer. Moreover, only intermolecular interactions were represented in the plots. It can be observed that in all cases the Se⋯N ChBs are characterized by a bond critical point (CP) and bond path connecting the N to the Se atoms. The bond CPs are represented as red spheres and bond paths as orange lines in Figure 4. Moreover, the reduced density gradient (RDG) isosurfaces were represented overlapped with the QTAIM analysis. The NCIplot analysis shows the existence of blue disk-shaped RDG isosurfaces that further characterize the Se⋯N contacts, thus confirming the attractive nature of the interaction. Interestingly, a yellow RDG isosurface is also observed connecting the blue disks that characterize the Se⋯N bonds, as is detailed in the enlargement given in Figure 4f, thus revealing a N⋯N repulsion (blue and green colors are used for attractive interactions and yellow and red for repulsive). In fact, in compound **8**, where the N⋯N distance is shorter, a bond CP and bond path interconnect both nitrogen atoms, further confirming the existence of a N⋯N interaction. The yellow color of the isosurface evidences the repulsive contribution of this contact that is obviously compensated by the Se⋯N attraction. Interestingly, the existence of the Se⋯π ChBs is also confirmed by both the QTAIM and NCIplot analyses. The shape and extension of the RDG isosurfaces in all compounds reveal that most of the π-cloud of the aromatic ring participates in the ChB. The dimerization energies range from −15.6 kcal/mol in **5** to −22.6 kcal/mol in compound **10**, which confirms the importance of these interactions in the solid state of the 1,2,4-selenodiazolium salts reported herein. In an effort to estimate the relative contribution of the Se⋯π interaction, we also computed the dimerization energies using mutated dimers. The mutation consists of the replacement of the aromatic ring (either phenyl or naphtyl) by a hydrogen atom. As a consequence, the symmetrically equivalent Se⋯π interactions in the dimers are not established, and the binding energy is drastically reduced (see values in red in Figure 4). The values range from −4.8 kcal/mol in **5** to −9.3 kcal/mol in compound **10**, thus revealing that the contribution of the Se⋯π is indeed dominant in these complexes, in line with the participation of the deeper σ-hole of selenium.

**Figure 5:**
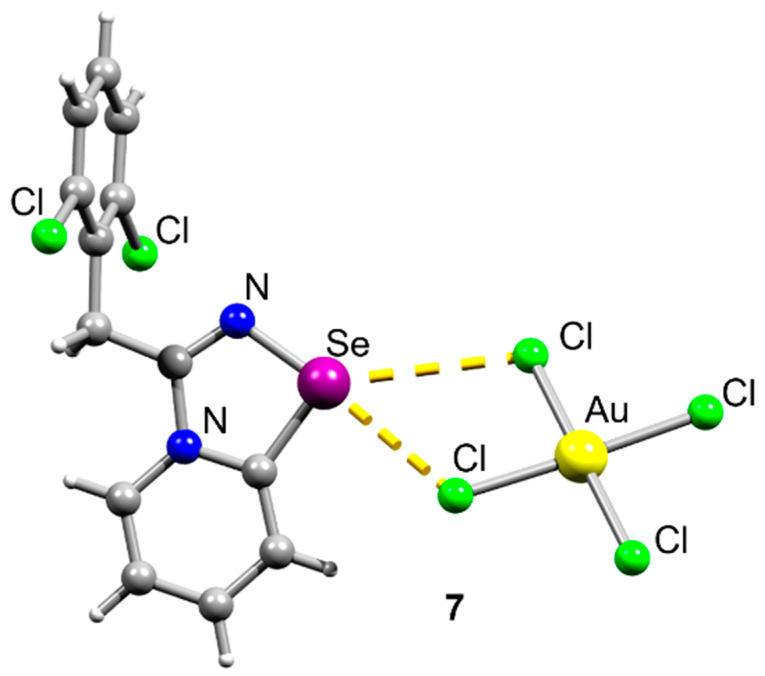
Ball-and-stick representation of crystal structure of **7**, demonstrating Se⋯Cl ChB interactions. Grey and light-grey spheres represent carbon and hydrogen, respectively.

Further, we were interested how introduction of electron deficient aryl substituent would affect the self-assembly of the corresponding selenodiazolium salts with weakly coordinating anion. Electron-deficient arenes are very weak Lewis bases, and their *η*^6^ metal complexes are very scarce. Thus, we expected that selenodiazolium cations carrying electron deficient aryls would not undergo supramolecular dimerization via two Se⋯N and Se⋯π ChB interactions. In order to verify this hypothesis, we prepared compound **7**, whose structure in the solid state was established by means of single-crystal X-ray crystallography (Figure 5).

As we expected, **7** did not feature Se⋯π ChB. This further demonstrate the predictable behavior of benzylic-substituted selenodiazolium salts towards self-aggregation via two Se⋯N and Se⋯π ChB interactions in the solid state and supports the theoretical analysis above that demonstrates the dominant role of the Se⋯π ChB in the self-assembled dimers.

## 3. Materials and Methods

### 3.1. General Remarks

All manipulations were carried out in air unless specified. All the reagents used in this study were obtained from the commercial sources (Aldrich, TCI-Europe, Strem, ABCR). Commercially available solvents were purified by conventional methods and distilled immediately prior to use. NMR spectra were recorded on a Bruker Avance neo-700; chemical shifts (*δ*) are given in ppm and coupling constants (J) in Hz. C, H, and N elemental analyses were carried out on a Euro EA 3028HT CHNS/O analyzer. Di(2-pyridyl)diselenide was prepared as reported earlier [39,40].

### 3.2. X-ray Crystal Structure Determination

The single-crystal X-ray diffraction data were collected on three-circle Bruker Smart APEX-II (**2**, **3**), Bruker D8 QUEST (**4**, **6**, **8**), and Bruker D8 Venture (**9**) diffractometers (graphite monochromator, *w* and *j* scanning mode) and on a four-circle Rigaku Synergy S diffractometer equipped with a HyPix6000HE area-detector (graphite monochromator, shutterless *ω*-scan mode) (**5**, **7**, **10**). For **2**–**4**, **6**, **8**, and **9**, the data were indexed and integrated using the SAINT program [53] and then scaled and corrected for absorption using the SADABS program [54]. For **5**, **7**, and **10**, the data were integrated and corrected for absorption by the *CrysAlisPro* program. For details, see Table S1 (Electronic Supporting Information). The structures were determined by direct methods and refined by full-matrix least-squares technique on *F*^2^ with anisotropic displacement parameters for non-hydrogen atoms. The hydrogen atoms in all compounds were placed in calculated positions and refined within riding model with fixed isotropic displacement parameters (*U*_iso_(H) = 1.5*U*_eq_(C) for the CH_3_-groups and 1.2*U*_eq_(C) for the other groups). All calculations were carried out using the SHELXTL program [55].

One molecule of MeOH in the unit cell (V = 232 A^3^ in one void) of **9** was found to be disordered over multiple positions and could therefore not be modelled satisfactorily. It was removed from the electron density map using the OLEX solvent mask command.

Crystallographic data for all investigated compounds were deposited with the Cambridge Crystallographic Data Center, CCDC 2220382-2220390. Copies of this information may be obtained free of charge from the Director, CCDC, 12 Union Road, Cambridge CHB2 1EZ, UK (Fax: +44-1223-336033; e-mail: deposit@ccdc.cam.ac.uk or www.ccdc.cam.ac.uk, accessed on 18 November 2022).

### 3.3. Computational Details

The single-point calculations of the self-assembled dimers of compounds **5**, **6**, **8**, **9**, and **10** were performed using the Turbomole 7.2 program [56]. The level of theory used was PBE0-D3/def2-TZVP [57,58,59,60] since it has been recently used for similar interactions [61]. The MEP surfaces were generated at the same level of theory and the 0.001 a.u. isosurface. The QTAIM [62] distribution of CPs and bond paths and NCIplot RDG isosurfaces [63] were plotted using the VMD program [63]. The following settings were used for the RDG plots: s = 0.45 a.u.; cut-off ρ = 0.04 a.u.; and color scale −0.025 a.u. ≤ sign(λ_2_)ρ ≤ 0.025 a.u.

### 3.4. Synthetic Procedures

**Synthesis of 2.** The solution of PhICl_2_ (21.6 mg, 0.08 mmol) and phenylacetonitrile (500 µL) in CH_2_Cl_2_ (3 mL) was added to 2,2′-dipyridyldiselenide (24.9 mg, 0.08 mmol) in CH_2_Cl_2_ (1 mL), and the reaction mixture was left without stirring for 5 h. After that, a solution was decanted from colorless crystalline precipitate, which was washed with Et_2_O (3 × 1 mL) and dried under vacuum. Yield: 45.6 mg (93%). ^1^H NMR (600 MHz, CD_3_OD) *δ* 9.46 (d, *J* = 6.8 Hz, 1H, H5), 8.98 (d, *J* = 8.7 Hz, 1H, H8), 8.39 (t, *J* = 8.4 Hz, 1H, H7), 7.98 (t, *J* = 7.0 Hz, 1H, H6), 7.42–7.24 (m, 5H, from Ph’s), 4.74 (s, 2H, CH_2_). ^13^C NMR (151 MHz, CD_3_OD) *δ* 170.6 (C3), 158.2 (C9), 140.3 (C5), 137.7 (C8), 127.9 (C7), 124.2 (C6), 134.1 (C from Ph’s), CH from Ph’s: 130.6, 130.2, 129.0; 38.4 (CH_2_). MS (ESI^+^), found: 275.0082 [M–Cl]^+^; calcd for C_13_H_11_N_2_Se: 275.0082.

**Synthesis of 3**. The solution of PhICl_2_ (15.6 mg, 0.06 mmol) and 2-(naphthalen-1-yl)acetonitrile (50 mg) in CH_2_Cl_2_ (3 mL) was added to 2,2′-dipyridyldiselenide (17.8 mg, 0.06 mmol) in CH_2_Cl_2_ (1 mL), and the reaction mixture was left without stirring for 5 h. After that, a solution was decanted from colorless crystalline precipitate, which was washed with Et_2_O (3 × 1 mL) and dried under vacuum. Yield: 39 mg (90%). ^1^H NMR (600 MHz, CD_3_OD) *δ* 9.62 (d, *J* = 6.7 Hz, 1H, H5), 8.98 (d, *J* = 8.7 Hz, 1H, H8), 8.49–8.42 (m, 1H, H7), 8.11–8.03 (m, 2H), 7.97–7.88 (m, 2H), 7.56–7.43 (m, 4H), 5.18 (s, 2H, CH_2_). ^13^C NMR (151 MHz, CD_3_OD) δ 170.3 (C3), 157.8 (C9), 140.2 (C5), 137.7 (C8), 135.5, 133.6, 130.9, 130.5, 129.8, 129.0, 127.7, 127.6, 127.1, 126.5, 124.9 (C7), 124.0 (C6), 36.4 (CH_2_). MS (ESI^+^), found: 325.0239 [M–Cl]^+^; calcd for C_17_H_13_N_2_Se: 325.0238.

**Synthesis of 4.** The solution of PhICl_2_ (22 mg, 0.08 mmol) and 2-(2,6-dichlorophenyl)acetonitrile (50 mg) in CH_2_Cl_2_ (3 mL) was added to 2,2′-dipyridyldiselenide (25 mg, 0.08 mmol) in CH_2_Cl_2_ (1 mL), and the reaction mixture was left without stirring for 5 h. After that, a solution was decanted from yellow crystalline precipitate, which was washed with Et_2_O (3 × 1 mL) and dried under vacuum. Yield: 47 mg (78%). ^1^H NMR (600 MHz, D_2_O) δ 9.59 (d, *J* = 6.9 Hz, 1H, H5), 8.85 (d, *J* = 8.6 Hz, 1H, H8), 8.47 (t, *J* = 8.3 Hz, 1H), 8.56–8.41 (m, 1H, H7), 8.19–8.05 (m, 1H, H6), 7.55 (d, J = 8.1 Hz, 2H), 7.42 (dd, J = 8.5, 7.8 Hz, 1H), 5.01 (s, 2H, CH_2_). ^13^C NMR (151 MHz, D_2_O) δ 168.3, 155.0, 134.0, 136.2, 135.9, 130.6, 129.4, 128.6, 126.1, 123.3, 33.7 (CH_2_).

**Synthesis of 5.** The solution of PhICl_2_ (28.6 mg, 0.08 mmol) and phenylacetonitrile (500 µL) in CH_2_Cl_2_ (3 mL) was added to 2,2′-dipyridyldiselenide (24.9 mg, 0.09 mmol) in CH_2_Cl_2_ (1 mL), and the reaction mixture was left without stirring for 5 h. After that, a solution was decanted from colorless crystalline precipitate, which was washed with Et_2_O (3 × 1 mL) and dried under vacuum. Addition of the MeOH solution (100 µL) of NaAuCl_4_ (50 mg) to MeOH solution of **2** (1 mL) resulted in the formation of yellow microcrystalline precipitate. Yield: 64 mg (65%). ^1^H NMR (600 MHz, Me_2_CO-*d_6_*) *δ* 9.74 (d, *J* = 6.8, 1H, H5), 9.16 (d, *J* = 8.6, 1H, H8), 8.61 (ddd, *J* = 8.5, 7.2, 1.1 Hz, 1H, H7), 8.23 (t, *J* = 7.0, 1H, H6), 7.52–7.36 (m, 5H), 4.94 (s, 2H, CH_2_). ^13^C NMR (151 MHz, Me_2_CO-*d_6_*) *δ* 169.7 (C3), 158.1 (C9), 140.6 (C5), 137.5 (C8), 130.5, 129.7, 128.6, 127.4 (C7), 124.2 (C6), 133.7 (C from Ph), 38.1 (CH_2_).

**Synthesis of 6.** The solution of PhICl_2_ (16.5 mg, 0.06 mmol) and 2-(naphthalen-1-yl)acetonitrile (50 mg) in CH_2_Cl_2_ (3 mL) was added to 2,2′-dipyridyldiselenide (19 mg, 0.06 mmol) in CH_2_Cl_2_ (1 mL), and the reaction mixture was left without stirring for 5 h. After that, a solution was decanted from colorless crystalline precipitate, which was washed with Et_2_O (3 × 1 mL) and dried under vacuum. Addition of the MeOH solution (100 µL) of NaAuCl_4_ (50 mg) to MeOH solution of **3** (1 mL) resulted in the formation of orange microcrystalline precipitate. Yield: 46 mg (58%). ^1^H NMR (600 MHz, Me_2_CO-*d_6_*) *δ* 9.95 (d, *J* = 6.8 Hz, 1H, H5), 9.18 (d, *J* = 8.7 Hz, 1H, H8), 8.66 (ddd, *J* = 8.4, 7.3, 0.9 Hz, 1H, H7), 8.31 (td, *J* = 7.0, 1.1 Hz, 1H, H6), 8.13 (d, *J* = 8.4 Hz, 1H), 8.01 (dd, *J* = 17.6, 8.2 Hz, 2H), 7.59–7.50 (m, 4H), 5.38 (s, 2H, CH_2_). ^13^C NMR (151 MHz, Me_2_CO-*d_6_*) δ 169.6 (C3), 157.8 (C9), 145.6, 140.6 (C5), 138.0 (C8), 135.0, 133.3, 130.4, 129.5, 129.1, 127.3, 126.9, 126.4, 125.3, 125.18 (C7), 124.1 (C7), 36.2 (CH_2_).

**Synthesis of 7.** The solution of PhICl_2_ (16.1 mg, 0.06 mmol) and 2-(2,6-dichlorophenyl)acetonitrile (50 mg) in CH_2_Cl_2_ (3 mL) was added to 2,2′-dipyridyldiselenide (18.5 mg, 0.06 mmol) in CH_2_Cl_2_ (1 mL), and the reaction mixture was left without stirring for 5 h. After that, a solution was decanted from yellow crystalline precipitate, which was washed with Et_2_O (3 × 1 mL) and dried under vacuum. Addition of the MeOH solution (100 µL) of NaAuCl_4_ (50 mg) to MeOH solution of **4** (1 mL) resulted in the formation of orange microcrystalline precipitate. Yield: 39 mg (48%). ^1^H NMR (600 MHz, Me_2_CO-*d_6_*) *δ* 10.01 (d, *J* = 6.8 Hz, 1H, H5), 9.21 (d, *J* = 8.6 Hz, 1H, H8), 8.67 (ddd, *J* = 8.4, 7.2, 1.0 Hz, 1H, H7), 8.38–8.32 (m, 1H, H6), 7.63–7.58 (m, 2H), 7.46 (dd, *J* = 8.6, 7.5 Hz, 1H), 5.19 (s, 2H, CH_2_). ^13^C NMR (151 MHz, Me_2_CO-*d_6_*) δ 155.2, 140.9, 137.8, 137.0, 131.7, 131.4, 129.7, 129.5, 127.6, 124.3, 34.6 (CH_2_).

**Synthesis of 8.** The solution of PhICl_2_ (22 mg, 0.08 mmol) and phenylacetonitrile (500 µL) in CH_2_Cl_2_ (3 mL) was added to 2,2′-dipyridyldiselenide (25 mg, 0.08 mmol) in CH_2_Cl_2_ (1 mL), and the reaction mixture was left without stirring for 5 h. After that, a solution was decanted from colorless crystalline precipitate, which was washed with Et_2_O (3 × 1 mL) and dried under vacuum. Addition of the MeOH solution (100 µL) of NaClO_4_ resulted in the formation of colorless microcrystalline precipitate. Addition of the MeOH solution (100 µL) of LiClO_4_ to MeOH solution of **2** (1 mL) resulted in the formation of colorless microcrystalline precipitate. Yield: 40 mg (67%). ^1^H NMR (600 MHz, Me_2_CO-*d_6_*) *δ* 9.73 (d, *J* = 6.8 Hz, 1H, H5), 9.14 (d, *J* = 8.6 Hz, 1H, H8), 8.59 (ddt, *J* = 8.5, 7.2, 1.0 Hz, 1H, H7), 8.20 (t, *J* = 6.9 Hz, 1H, H6), 7.49–7.38 (m, 5H), 4.92 (s, 2H, CH_2_). ^13^C NMR (151 MHz, Me_2_CO-*d_6_*) *δ* 158.2, 140.5, 137.7, 133.9, 130.5, 129.7, 128.9, 128.5, 127.4, 124.1, 38.1 (CH_2_).

**Synthesis of 9.** The solution of PhICl_2_ (20.3 mg, 0.06 mmol) and 2-(naphthalen-1-yl)acetonitrile (50 mg) in CH_2_Cl_2_ (3 mL) was added to 2,2′-dipyridyldiselenide (17.8 mg, 0.06 mmol) in CH_2_Cl_2_ (1 mL), and the reaction mixture was left without stirring for 5 h. After that, a solution was decanted from colorless crystalline precipitate, which was washed with Et_2_O (3 × 1 mL) and dried under vacuum. Addition of the MeOH solution (100 µL) of LiClO_4_ to MeOH solution of **3** (1 mL) resulted in the formation of colorless microcrystalline precipitate. Yield: 32 mg (61%). ^1^H NMR (600 MHz, Me_2_CO-*d_6_*) *δ* 9.86 (d, *J* = 6.8 Hz, 1H, H5), 9.07 (d, *J* = 8.7 Hz, 1H, H8), 8.62–8.55 (m, 1H, H7), 8.22 (td, *J* = 7.0 Hz, 1H, H6), 8.10 (d, *J* = 8.5 Hz, 1H), 8.01–7.92 (m, 2H), 7.57–7.44 (m, 4H), 5.30 (s, 2H, CH_2_). ^13^C NMR (151 MHz, Me_2_CO-*d_6_*) *δ* 157.7, 140.4, 137.8, 134.9, 133.4, 130.4, 129.6, 129.4, 129.0, 127.1, 127.0, 126.9, 126.48, 125.1, 123.9, 100.9, 35.2 (CH_2_).

**Synthesis of 10.** The solution of PhICl_2_ (18 mg, 0.07 mmol) and 2-(naphthalen-1-yl)acetonitrile (50 mg) in CH_2_Cl_2_ (3 mL) was added to 2,2′-dipyridyldiselenide (22 mg, 0.07 mmol) in CH_2_Cl_2_ (1 mL), and the reaction mixture was left without stirring for 5 h. After that, a solution was decanted from colorless crystalline precipitate, which was washed with Et_2_O (3 × 1 mL) and dried under vacuum. Addition of the saturated MeOH solution of tetrabutylammonium hexafluorophosphate (100 µL) to MeOH solution of **3** (1 mL) resulted in the formation of microcrystalline precipitate. Yield: 35 mg (53%). ^1^H NMR (600 MHz, Me_2_CO-*d_6_*) *δ* 9.92 (d, *J* = 6.9 Hz, 1H, H5), 9.15 (d, *J* = 8.7 Hz, 1H, H8), 8.67–8.60 (m, 1H, H7), 8.30–8.25 (m, 1H, H6), 8.13 (d, *J* = 8.6, 1.0 Hz, 1H), 8.03–7.98 (m, 2H), 7.59–7.49 (m, 4H), 5.36 (s, 2H, CH_2_). ^13^C NMR (151 MHz, Me_2_CO-*d_6_*) δ 169.5, 157.9, 140.7, 138.0, 135.0, 133.4, 130.5, 129.6, 129.2, 127.7, 127.3, 127.2, 126.9, 126.5, 125.2, 124.1, 36.2 (CH_2_).

## 4. Conclusions

We have synthesized a series of benzylic-substituted 1,2,4-selenodiazolium salts with weakly binding anions that promote the formation self-assembled dimers with the recurrent Se_2_N_2_ supramolecular motif. The dimers are further supported by two symmetrically equivalent selenium⋯arene ChB interactions. The Se⋯N and Se⋯π ChB interactions have been studied theoretically, and we disclosed the fundamental role of the Se⋯π interactions, which are stronger than the Se⋯N interactions observed in the Se_2_N_2_ motifs. The DFT analysis demonstrates that the stronger nature of the Se–π interactions is due to the participation of the more positive σ-hole and to the existence of a repulsive N⋯N interaction in the Se_2_N_2_ motif. The relevance of the Se⋯π interaction is further supported by the fact that compound **7** does not exhibit the Se⋯π interaction in the solid state due to the presence of electron withdrawing groups in the phenyl ring.

Importantly, the herein-described strategy can be employed for reliable supramolecular dimerization of 1,2,4-selenodiazolium cations in the solid state. This supramolecular synthon can be employed in supramolecular engineering for the creation of SOFs with programmable structures and properties.

## Supplementary Materials

The supporting information can be downloaded at: https://www.mdpi.com/article/10.3390/ijms232314973/s1.

## References

[1] O’Keeffe M., Yaghi O.M. (2005). Reticular chemistry-present and future prospects-Introduction. J. Solid State Chem..

[2] Li H., Eddaoudi M., O’Keeffe M., Yaghi O.M. (1999). Design and synthesis of an exceptionally stable and highly porous metal-organic framework. Nature.

[3] Slater A.G., Cooper A.I. (2015). Function-led design of new porous materials. Science.

[4] Wang B., Lin R.-B., Zhang Z., Xiang S., Chen B. (2020). Hydrogen-Bonded Organic Frameworks as a Tunable Platform for Functional Materials. J. Am. Chem. Soc..

[5] Desiraju G.R. (1995). Supramolecular Synthons in Crystal Engineering—A New Organic Synthesis. Angew. Chem. Int. Ed..

[6] Tskhovrebov A.G., Novikov A.S., Odintsova O.V., Mikhaylov V.N., Sorokoumov V.N., Serebryanskaya T.V., Starova G.L. (2019). Supramolecular polymers derived from the PtII and PdII schiff base complexes via C(sp2)–H … Hal hydrogen bonding: Combined experimental and theoretical study. J. Organomet. Chem..

[7] Repina O.V., Novikov A.S., Khoroshilova O.V., Kritchenkov A.S., Vasin A.A., Tskhovrebov A.G. (2020). Lasagna-like supramolecular polymers derived from the PdII osazone complexes via C(sp2)–H⋯Hal hydrogen bonding. Inorg. Chim. Acta.

[8] Mikhaylov V.N., Sorokoumov V.N., Novikov A.S., Melnik M.V., Tskhovrebov A.G., Balova I.A. (2020). Intramolecular hydrogen bonding stabilizes trans-configuration in a mixed carbene/isocyanide PdII complexes. J. Organomet. Chem..

[9] Scheiner S. (2013). The Pnicogen Bond: Its Relation to Hydrogen, Halogen, and Other Noncovalent Bonds. Acc. Chem. Res..

[10] Legon A.C. (2010). The halogen bond: An interim perspective. Phys. Chem. Chem. Phys..

[11] Murray J.S., Lane P., Clark T., Riley K.E., Politzer P. (2012). σ-Holes, π-holes and electrostatically-driven interactions. J. Mol. Model..

[12] Metrangolo P., Neukirch H., Pilati T., Resnati G. (2005). Halogen Bonding Based Recognition Processes:  A World Parallel to Hydrogen Bonding. Acc. Chem. Res..

[13] Nemec V., Fotović L., Vitasović T., Cinčić D. (2019). Halogen bonding of the aldehyde oxygen atom in cocrystals of aromatic aldehydes and 1,4-diiodotetrafluorobenzene. CrystEngComm.

[14] Cavallo G., Metrangolo P., Milani R., Pilati T., Priimagi A., Resnati G., Terraneo G. (2016). The Halogen Bond. Chem. Rev..

[15] Wang W., Ji B., Zhang Y. (2009). Chalcogen Bond: A Sister Noncovalent Bond to Halogen Bond. J. Phys. Chem. A.

[16] Benz S., López-Andarias J., Mareda J., Sakai N., Matile S. (2017). Catalysis with Chalcogen Bonds. Angew. Chem. Int. Ed..

[17] Riwar L.-J., Trapp N., Root K., Zenobi R., Diederich F. (2018). Supramolecular Capsules: Strong versus Weak Chalcogen Bonding. Angew. Chem. Int. Ed..

[18] Mahmudov K.T., Kopylovich M.N., Guedes da Silva M.F.C., Pombeiro A.J.L. (2017). Chalcogen bonding in synthesis, catalysis and design of materials. Dalt. Trans..

[19] Garrett G.E., Gibson G.L., Straus R.N., Seferos D.S., Taylor M.S. (2015). Chalcogen Bonding in Solution: Interactions of Benzotelluradiazoles with Anionic and Uncharged Lewis Bases. J. Am. Chem. Soc..

[20] De Vleeschouwer F., Denayer M., Pinter B., Geerlings P., De Proft F. (2018). Characterization of chalcogen bonding interactions via an in-depth conceptual quantum chemical analysis. J. Comput. Chem..

[21] Price S.L., Stone A.J., Lucas J., Rowland R.S., Thornley A.E. (1994). The Nature of -Cl.cntdot..cntdot..cntdot.Cl- Intermolecular Interactions. J. Am. Chem. Soc..

[22] Benz S., Poblador-Bahamonde A.I., Low-Ders N., Matile S. (2018). Catalysis with Pnictogen, Chalcogen, and Halogen Bonds. Angew. Chem. Int. Ed..

[23] Pascoe D.J., Ling K.B., Cockroft S.L. (2017). The Origin of Chalcogen-Bonding Interactions. J. Am. Chem. Soc..

[24] Li Q., Li R., Zhou Z., Li W., Cheng J. (2012). S⋯X halogen bonds and H⋯X hydrogen bonds in H2CS–XY (XY = FF, ClF, ClCl, BrF, BrCl, and BrBr) complexes: Cooperativity and solvent effect. J. Chem. Phys..

[25] Eliseeva A.A., Ivanov D.M., Novikov A.S., Kukushkin V.Y. (2019). Recognition of the π-hole donor ability of iodopentafluorobenzene—A conventional σ-hole donor for crystal engineering involving halogen bonding. CrystEngComm.

[26] Nelyubina Y.V., Antipin M.Y., Lyssenko K.A. (2011). Extremely short halogen bond: The nature and energy of iodine–oxygen interactions in crystalline iodic acid. Mendeleev Commun..

[27] Tsirelson V.G., Zhou P.F., Tang T.-H., Bader R.F.W. (1995). Topological definition of crystal structure: Determination of the bonded interactions in solid molecular chlorine. Acta Crystallogr. Sect. A.

[28] Brezgunova M.E., Aubert E., Dahaoui S., Fertey P., Lebègue S., Jelsch C., Ángyán J.G., Espinosa E. (2012). Charge Density Analysis and Topological Properties of Hal3-Synthons and Their Comparison with Competing Hydrogen Bonds. Cryst. Growth Des..

[29] Bauzá A., Frontera A. (2017). On the Importance of Halogen–Halogen Interactions in the Solid State of Fullerene Halides: A Combined Theoretical and Crystallographic Study. Crystals.

[30] Li H., Lu Y., Liu Y., Zhu X., Liu H., Zhu W. (2012). Interplay between halogen bonds and π–π stacking interactions: CSD search and theoretical study. Phys. Chem. Chem. Phys..

[31] Eckstein B.J., Brown L.C., Noll B.C., Moghadasnia M.P., Balaich G.J., McGuirk C.M. (2021). A Porous Chalcogen-Bonded Organic Framework. J. Am. Chem. Soc..

[32] Berionni G., Pégot B., Marrot J., Goumont R. (2009). Supramolecular association of 1,2,5-chalcogenadiazoles: An unexpected self-assembled dissymetric [Se⋯N]2 four-membered ring. CrystEngComm.

[33] Alfuth J., Zadykowicz B., Sikorski A., Połoński T., Eichstaedt K., Olszewska T. (2020). Effect of Aromatic System Expansion on Crystal Structures of 1,2,5-Thia- and 1,2,5-Selenadiazoles and Their Quaternary Salts: Synthesis, Structure, and Spectroscopic Properties. Materials.

[34] Ho P.C., Wang J.Z., Meloni F., Vargas-Baca I. (2020). Chalcogen bonding in materials chemistry. Coord. Chem. Rev..

[35] Risto M., Reed R.W., Robertson C.M., Oilunkaniemi R., Laitinen R.S., Oakley R.T. (2008). Self-association of the N-methyl benzotellurodiazolylium cation: Implications for the generation of super-heavy atom radicals. Chem. Commun..

[36] Kumar V., Xu Y., Bryce D.L. (2020). Double Chalcogen Bonds: Crystal Engineering Stratagems via Diffraction and Multinuclear Solid-State Magnetic Resonance Spectroscopy. Chem.-A Eur. J..

[37] Ams M.R., Trapp N., Schwab A., Milić J.V., Diederich F. (2019). Chalcogen Bonding “2S–2N Squares” versus Competing Interactions: Exploring the Recognition Properties of Sulfur. Chem.-A Eur. J..

[38] Tiekink E.R.T. (2021). Supramolecular aggregation patterns featuring Se⋯N secondary-bonding interactions in mono-nuclear selenium compounds: A comparison with their congeners. Coord. Chem. Rev..

[39] Khrustalev V.N., Grishina M.M., Matsulevich Z.V., Lukiyanova J.M., Borisova G.N., Osmanov V.K., Novikov A.S., Kirichuk A.A., Borisov A.V., Solari E. (2021). Novel cationic 1,2,4-selenadiazoles: Synthesis via addition of 2-pyridylselenyl halides to unactivated nitriles, structures and four-center Se⋯N contacts. Dalt. Trans..

[40] Grudova M.V., Khrustalev V.N., Kubasov A.S., Strashnov P.V., Matsulevich Z.V., Lukiyanova J.M., Borisova G.N., Kritchenkov A.S., Grishina M.M., Artemjev A.A. (2022). Adducts of 2-Pyridylselenenyl Halides and Nitriles as Novel Supramolecular Building Blocks: Four-Center Se⋯N Chalcogen Bonding versus Other Weak Interactions. Cryst. Growth Des..

[41] Buslov I.V., Novikov A.S., Khrustalev V.N., Grudova M.V., Kubasov A.S., Matsulevich Z.V., Borisov A.V., Lukiyanova J.M., Grishina M.M., Kirichuk A.A. (2021). 2-Pyridylselenenyl versus 2-Pyridyltellurenyl Halides: Symmetrical Chalcogen Bonding in the Solid State and Reactivity towards Nitriles. Symmetry.

[42] Grudova M.V., Kubasov A.S., Khrustalev V.N., Novikov A.S., Kritchenkov A.S., Nenajdenko V.G., Borisov A.V., Tskhovrebov A.G. (2022). Exploring Supramolecular Assembly Space of Cationic 1,2,4-Selenodiazoles: Effect of the Substituent at the Carbon Atom and Anions. Molecules.

[43] Artemjev A.A., Novikov A.P., Burkin G.M., Sapronov A.A., Kubasov A.S., Nenajdenko V.G., Khrustalev V.N., Borisov A.V., Kirichuk A.A., Kritchenkov A.S. (2022). Towards Anion Recognition and Precipitation with Water-Soluble 1,2,4-Selenodiazolium Salts: Combined Structural and Theoretical Study. Int. J. Mol. Sci..

[44] Egorov A.R., Khubiev O., Rubanik V.V., Rubanik V.V., Lobanov N.N., Savilov S.V., Kirichuk A.A., Kritchenkov I.S., Tskhovrebov A.G., Kritchenkov A.S. (2022). The first selenium containing chitin and chitosan derivatives: Combined synthetic, catalytic and biological studies. Int. J. Biol. Macromol..

[45] Egorov A.R., Yagafarov N.Z., Artemjev A.A., Khubiev O., Medjbour B., Kozyrev V.A., Donovan Sikaona N., Tsvetkova O.I., Rubanik V.V., Rubanik V.V. (2022). Synthesis and in vitro antifungal activity of selenium-containing chitin derivatives. Mendeleev Commun..

[46] Shikhaliyev N.Q., Ahmadova N.E., Gurbanov A.V., Maharramov A.M., Mammadova G.Z., Nenajdenko V.G., Zubkov F.I., Mahmudov K.T., Pombeiro A.J.L. (2018). Tetrel, halogen and hydrogen bonds in bis(4-((E)-(2,2-dichloro-1-(4-substitutedphenyl)vinyl)diazenyl)phenyl)methane dyes. Dye. Pigment..

[47] Shikhaliyev N.G., Maharramov A.M., Bagirova K.N., Suleymanova G.T., Tsyrenova B.D., Nenajdenko V.G., Novikov A.S., Khrustalev V.N., Tskhovrebov A.G. (2021). Supramolecular organic frameworks derived from bromoaryl-substituted dichlorodiazabutadienes via Cl⋯Br halogen bonding. Mendeleev Commun..

[48] Nenajdenko V.G., Shastin A.V., Gorbachev V.M., Shorunov S.V., Muzalevskiy V.M., Lukianova A.I., Dorovatovskii P.V., Khrustalev V.N. (2017). Copper-Catalyzed Transformation of Hydrazones into Halogenated Azabutadienes, Versatile Building Blocks for Organic Synthesis. ACS Catal..

[49] Tskhovrebov A.G., Vasileva A.A., Goddard R., Riedel T., Dyson P.J., Mikhaylov V.N., Serebryanskaya T.V., Sorokoumov V.N., Haukka M. (2018). Palladium(II)-Stabilized Pyridine-2-Diazotates: Synthesis, Structural Characterization, and Cytotoxicity Studies. Inorg. Chem..

[50] Liu Y., Varava P., Fabrizio A., Eymann L.Y.M., Tskhovrebov A.G., Planes O.M., Solari E., Fadaei-Tirani F., Scopelliti R., Sienkiewicz A. (2019). Synthesis of aminyl biradicals by base-induced Csp3–Csp3 coupling of cationic azo dyes. Chem. Sci..

[51] Nenajdenko V.G., Shikhaliyev N.G., Maharramov A.M., Bagirova K.N., Suleymanova G.T., Novikov A.S., Khrustalev V.N., Tskhovrebov A.G. (2020). Halogenated Diazabutadiene Dyes: Synthesis, Structures, Supramolecular Features, and Theoretical Studies. Molecules.

[52] Tskhovrebov A.G., Novikov A.S., Tupertsev B.S., Nazarov A.A., Antonets A.A., Astafiev A.A., Kritchenkov A.S., Kubasov A.S., Nenajdenko V.G., Khrustalev V.N. (2021). Azoimidazole Gold(III) Complexes: Synthesis, Structural Characterization and Self-Assembly in the Solid State. Inorganica Chim. Acta.

[53] (2018). Bruker, SAINT.

[54] Krause L., Herbst-Irmer R., Sheldrick G.M., Stalke D. (2015). Comparison of silver and molybdenum microfocus X-ray sources for single-crystal structure determination. J. Appl. Crystallogr..

[55] Sheldrick G.M. (2015). Crystal structure refinement with SHELXL. Acta Crystallogr. Sect. C Struct. Chem..

[56] Ahlrichs R., Bär M., Häser M., Horn H., Kölmel C. (1989). Electronic structure calculations on workstation computers: The program system turbomole. Chem. Phys. Lett..

[57] Adamo C., Barone V. (1999). Toward reliable density functional methods without adjustable parameters: The PBE0 model. J. Chem. Phys..

[58] Grimme S., Antony J., Ehrlich S., Krieg H. (2010). A consistent and accurate ab initio parametrization of density functional dispersion correction (DFT-D) for the 94 elements H-Pu. J. Chem. Phys..

[59] Weigend F., Ahlrichs R. (2005). Balanced basis sets of split valence, triple zeta valence and quadruple zeta valence quality for H to Rn: Design and assessment of accuracy. Phys. Chem. Chem. Phys..

[60] Weigend F. (2006). Accurate Coulomb-fitting basis sets for H to Rn. Phys. Chem. Chem. Phys..

[61] Gomila R.M., Frontera A. (2022). Matere Bonds vs. Multivalent Halogen and Chalcogen Bonds: Three Case Studies. Molecules.

[62] Gomila R.M., Frontera A. (2022). Crystallographic and Theoretical Study of Osme Bonds in Nitrido-Osmium(VI) Complexes. Inorganics.

[63] Bader R.F.W. (1991). A Quantum Theory of Molecular Structure and Its Applications. Chem. Rev..

