# Supplementary material for: Robust Supramolecular Dimers Derived from Benzylic-Substituted 1,2,4-Selenodiazolium Salts Featuring Selenium⋯π Chalcogen Bonding
Source: Int J Mol Sci. 2022 Nov 29;23(23):14973. doi: 10.3390/ijms232314973 (PMC9740427; doi:10.3390/ijms232314973)
Supplement: Supplementary file 1 [file ijms-23-14973-s001.zip › ijms-2075277-supplementary.pdf]

Table S1. Crystal data and structure refinement for 2-10.

Identification code	2	3	4
Empirical formula	$C_7H_{6.5}Cl_{1.5}NSe_{0.5}$	$C_{17}H_{13}ClN_2Se$	$C_{13}H_9Cl_3N_2Se$
Formula weight	197.29	359.70	378.53
Temperature/K	150.00	100	100.15
Crystal system	monoclinic	tetragonal	triclinic
Space group	$P2_1/m$	$P4_1$	P-1
a/Å	9.0688(9)	7.9243(6)	7.86270(10)
b/Å	6.9679(6)	7.9243(6)	8.5138(2)
c/Å	12.7072(10)	22.6512(12)	11.0901(2)
α/°	90	90	78.3468(6)
β/°	95.281(4)	90	88.0530(6)
γ/°	90	90	69.5161(6)
Volume/$Å^3$	799.57(12)	1422.4(2)	680.57(2)
Z	4	4	2
$\rho_{calc}g/cm^3$	1.639	1.680	1.847
μ/mm^{-1}	2.840	2.821	3.332
F(000)	392.0	720.0	372.0
Radiation	MoKα (λ = 0.71073)	MoKα (λ = 0.71073)	MoKα (λ = 0.71073)
2Θ range for data collection/°	5.296 to 60.146	5.14 to 53.978	3.752 to 65.286
Reflections collected	5664	15269	17088
Independent reflections	2293 [R_{int} = 0.0280, R_{sigma} = 0.0382]	3037 [R_{int} = 0.0457, R_{sigma} = 0.0427]	4970 [R_{int} = 0.0309, R_{sigma} = 0.0313]
Data/restraints/parameters	2293/0/115	3037/1/190	4970/0/173
Goodness-of-fit on F^2	1.061	1.041	1.025
Final R indexes [I>=2σ (I)]	R_1 = 0.0268, wR_2 = 0.0565	R_1 = 0.0214, wR_2 = 0.0458	R_1 = 0.0235, wR_2 = 0.0511

Final R indexes [all data]	R_1 = 0.0334, wR_2 = 0.0602	R_1 = 0.0231, wR_2 = 0.0464	R_1 = 0.0296, wR_2 = 0.0537
Identification code	5	6	7
Empirical formula	$C_{13}H_{11}N_2Cl_4SeAu$	$C_{17}H_{13}AuCl_4N_2Se$	$C_{13}H_9AuCl_6N_2Se$
Formula weight	612.96	663.02	681.85
Temperature/K	100.15	100.15	100.15
Crystal system	monoclinic	monoclinic	monoclinic
Space group	$P2_1/n$	$P2_1/n$	$P2_1/c$
a/Å	13.2183(3)	12.7620(5)	7.84726(5)
b/Å	10.30381(19)	11.5911(4)	31.83002(11)
c/Å	13.2745(3)	13.1164(5)	7.92815(6)
α/°	90	90	90
β/°	110.170(3)	105.5418(8)	112.0285(7)
γ/°	90	90	90
Volume/$Å^3$	1697.10(7)	1869.31(12)	1835.71(2)
Z	4	4	4
$\rho_{calc}g/cm^3$	2.399	2.356	2.467
μ/mm^{-1}	11.435	10.392	25.355
F(000)	1136.0	1240.0	1264.0
Radiation	MoKα (λ = 0.71073)	MoKα (λ = 0.71073)	CuKα (λ = 1.54184)
2Θ range for data collection/°	5.13 to 63.404	4.768 to 65.676	5.552 to 155.488
Reflections collected	30962	49613	195254
Independent reflections	4943 [R_{int} = 0.0382, R_{sigma} = 0.0262]	6901 [R_{int} = 0.0340, R_{sigma} = 0.0196]	3867 [R_{int} = 0.1391, R_{sigma} = 0.0226]
Data/restraints/parameters	4943/0/191	6901/0/227	3867/0/209
Goodness-of-fit on F^2	1.086	1.094	1.093
Final R indexes [I>=2σ (I)]	R_1 = 0.0189, wR_2 = 0.0384	R_1 = 0.0255, wR_2 = 0.0519	R_1 = 0.0373, wR_2 = 0.1017
Final R indexes [all data]	R_1 = 0.0244, wR_2 = 0.0394	R_1 = 0.0310, wR_2 = 0.0537	R_1 = 0.0373, wR_2 = 0.1017

Identification code	8	9	10
Empirical formula	$C_{13}H_{11}ClN_2O_4Se$	$C_{17}H_{13}ClN_2O_4Se$	$C_{69}H_{56}F_{24}N_8OP_4Se_4$
Formula weight	373.65	423.70	1908.93
Temperature/K	100.15	100.00	103.15
Crystal system	monoclinic	monoclinic	monoclinic
Space group	$P2_1/n$	$P2_1/n$	$P2_1/n$
a/Å	10.7064(2)	14.642(6)	14.9255(4)
b/Å	11.4972(2)	13.984(10)	14.1500(4)
c/Å	12.0482(2)	17.319(8)	17.2098(4)
α/°	90	90	90
β/°	107.1724(5)	91.858(15)	92.295(2)
γ/°	90	90	90
Volume/$Å^3$	1416.94(4)	3544(3)	3631.72(16)
Z	4	8	2
ρ_{calc}g/cm^3	1.752	1.588	1.746
μ/mm^{-1}	2.855	2.294	4.262
F(000)	744.0	1696.0	1892.0
Radiation	MoKα (λ = 0.71073)	MoKα (λ = 0.71073)	CuKα (λ = 1.54184)
2Θ range for data collection/°	5.712 to 65.168	3.586 to 54.998	7.69 to 156.544
Reflections collected	50063	17580	46447
Independent reflections	5138 [R_{int} = 0.0226, R_{sigma} = 0.0111]	8081 [R_{int} = 0.0443, R_{sigma} = 0.0780]	7670 [R_{int} = 0.0976, R_{sigma} = 0.0480]
Data/restraints/parameters	5138/0/191	8081/16/439	7670/0/507
Goodness-of-fit on F^2	1.040	1.034	1.144
Final R indexes [I>=2σ (I)]	R_1 = 0.0165, wR_2 = 0.0434	R_1 = 0.0694, wR_2 = 0.1496	R_1 = 0.0864, wR_2 = 0.2162
Final R indexes [all data]	R_1 = 0.0183, wR_2 = 0.0444	R_1 = 0.1015, wR_2 = 0.1629	R_1 = 0.0894, wR_2 = 0.2183
